# A Projection Integration Method for X-ray Collimator Design

**DOI:** 10.7759/cureus.64482

**Published:** 2024-07-13

**Authors:** Tong Chen

**Affiliations:** 1 Research and Development, Zap Surgical Systems, San Carlos, USA

**Keywords:** beam penumbra, srs, x-ray profile, small field, collimator

## Abstract

One of the recent trends in radiation therapy is to increase conformal and accurate dose delivery such as in stereotactic radiosurgery (SRS). Treating small lesions and brain disorders requires the accurate placement of small radiation fields deep inside the human cranium. To design a collimator meeting these requirements, a new numerical concept was developed, which is presented here.

The algorithm proposed here can generate beam profiles of plural collimation apertures and arbitrary initial beam spot distributions in a time-efficient method. It is an ideal tool to optimize collimator design for penumbra, dose rate, and field size.

The intensity of the source beam spot is divided into slices, and each slice is projected onto the treatment plane at the isocenter through the collimator apertures. The illuminated field range and intensity are determined by geometry and the intensity of that slice of beam source, respectively. By integrating the projected intensity across all the slices of the source profile, the profile on the treatment plane is obtained.

The algorithm is used to generate beam profiles of a conical pencil beam collimator system and compare them to the Monte Carlo simulation as well as measurements. It can also be used to demonstrate the impact of collimator shape on the beam penumbra, dose rate, and field size.

The projection integration method provides a quick and informative tool for collimator design. The results were validated with the Monte Carlo simulation and measurements. This method was demonstrated to be effective for optimizing beam characteristics.

## Introduction

The development of modern radiation therapy techniques requires ever-increasing beam deposition accuracy and a more comprehensive approach to collimator design. This holds particularly true for stereotactic radiosurgery (SRS) treatment with the application of very small fields. To generate such beams, the collimator design needs to be carefully optimized so that the desired beam characteristics are achieved.

Typical collimator design is based on simple geometry projection, ray tracing, or a Monte Carlo simulation [1]. The ray tracing method is more time-efficient than the Monte Carlo simulation but it is performed as a lengthy interactive process [2,3]. When the radiation field is very small, particularly for long collimation channels, the penumbra and field size modeling become complex and difficult.

The Monte Carlo simulation provides the most comprehensive information for a collimation system. It accounts for all relevant physics phenomena and interactions such as scattering and penetration (leakage) and transports the full phase space through the system. However, any Monte Carlo simulation is computationally heavy. To achieve adequate statistics, long CPU times are needed. That makes Monte Carlo difficult for design and optimization.

The method presented here represents a practical solution for a fast yet accurate calculation of individual beam profiles iteratively, resulting in optimized time-efficient collimator design. Unlike Monte Carlo simulations, beam profile computations are generated almost instantly instead of over many hours [1], depending on the type of CPU or GPU used. On the other hand, the presented projection integration method can be completed in as little time as a second. This enables fast iteration for optimizations and lowers the threshold for innovative multi-iterative collimator design explorations.

The projection integration method takes a different approach, i.e., the transport of the beam phase space emittance through the collimation system [4]. In beam physics, emittance is a property of a beam that represents a collective of many particles. It refers to the area occupied by the beam in a position-and-momentum phase space. Rather than tracking individual particles, this method projects areas of phase space that contain information about a large number of particles and may include initial beam spot intensity profiles. As a result, the computation requirement is greatly reduced, and the result does not depend on event statistics as is the case for the Monte Carlo and ray tracing methods. By applying the projection method, the entire beam profile is generated at the treatment plane. From the profile, one can analyze the field size and penumbra and estimate the dose rate. Due to the inherent method efficiency, it presents a perfect solution for optimizing collimator designs.

## Technical report

**Figure 1:**
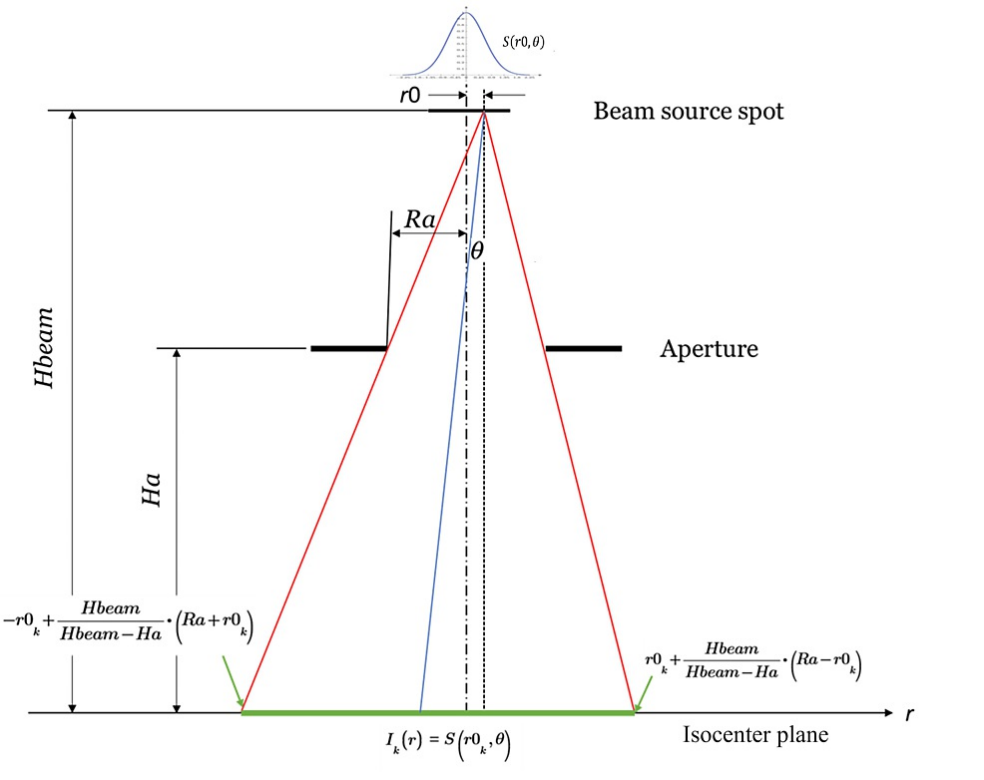
Collimator system schematic

The projection integration method divides the initial (or focus) beam spot into multiple slices (Figure 1). Each slice is defined by its position, intensity, and angular distribution. The slice width should be narrow enough to be approximated as a point source. This point source is projected through the system, limited by the collimators, onto the target plane with the intensity of the slice (air attenuation is ignored). By integrating the projected intensity on the treatment plane over all the slices, the final beam profile is obtained.

The basic schematic of the algorithm as shown in Figure 1. Slice by slice, the beam spot intensity *S* is analyzed sequentially. From its position and the collimator aperture, one can find the left edge and right edge of the illuminated field at the isocenter plane geometrically:

$EdgeLeft_{k}=-r0_{k}+\frac{Hbeam}{Hbeam-Ha}(Ra+r0_{k})$

$EdgeRight_{k}=r0_{k}+\frac{Hbeam}{Hbeam-Ha}(Ra-r0_{k})$,

where $r0_{k}$ is the *k*th slice radius of the spot. $Hbeam$ is the distance of the beam spot from the treatment plane. $Ha$ and $Ra$ are the height and radius of the aperture, respectively.

Between the two edges, the beam intensity is:

  $I_{k}(r)=S(r0_{k},\theta )$ , $-EdgeLeft_{k}&lt; r&lt; EdgeRight_{k}$ .

Beyond the edges, the intensity is set to zero.

**Figure 2:**
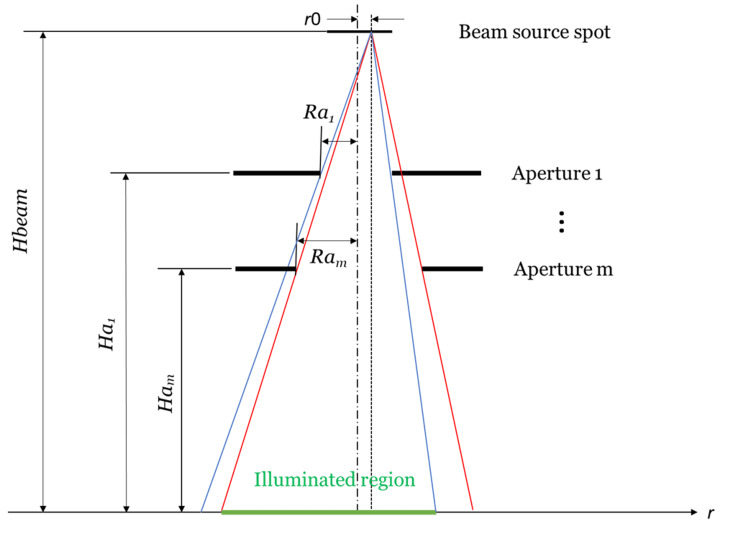
Collimator system with multiple apertures

When there are multiple collimation levels, as is typical for medical linear accelerators, the field edges for each level have to be calculated individually, as shown in Figure 2. The smallest value edge from both sides has to be chosen and the intensity between them has to be filled. This method can be expanded to more complex collimators and more than two levels of collimation.

The final step consists of integrating the intensities from each slice, so the total profile in the treatment plane is obtained:

$Profile(r)=\sum_{k}^{}I_{k}(r)$

Example and results

To demonstrate the method, a simple conical collimation system is programmed by PTC Mathcad [5], an engineering mathematical calculation software, to produce the profile at the treatment plane. The program starts with an initial beam intensity profile, e.g.,* *a Gaussian distribution, assigning the intensity of each slice of the initial beam at $r0$. The *k*th slice is taken, the $EdgeLeft_{k}$and $EdgeRight_{k}$ of every aperture are calculated, and the minimum of those $EdgeLeft_{k}$and $EdgeRight_{k}$ are taken to determine the range which the *k*th slice illuminated in the treatment plane (the green area as shown in Figure 2). The *k*th slice intensity value within the illuminated range and zero for the outside range are assigned. Finally, the intensity through all slices (*k*) along the treatment plane (*r*) is summed up and the profile is obtained.

**Figure 3:**
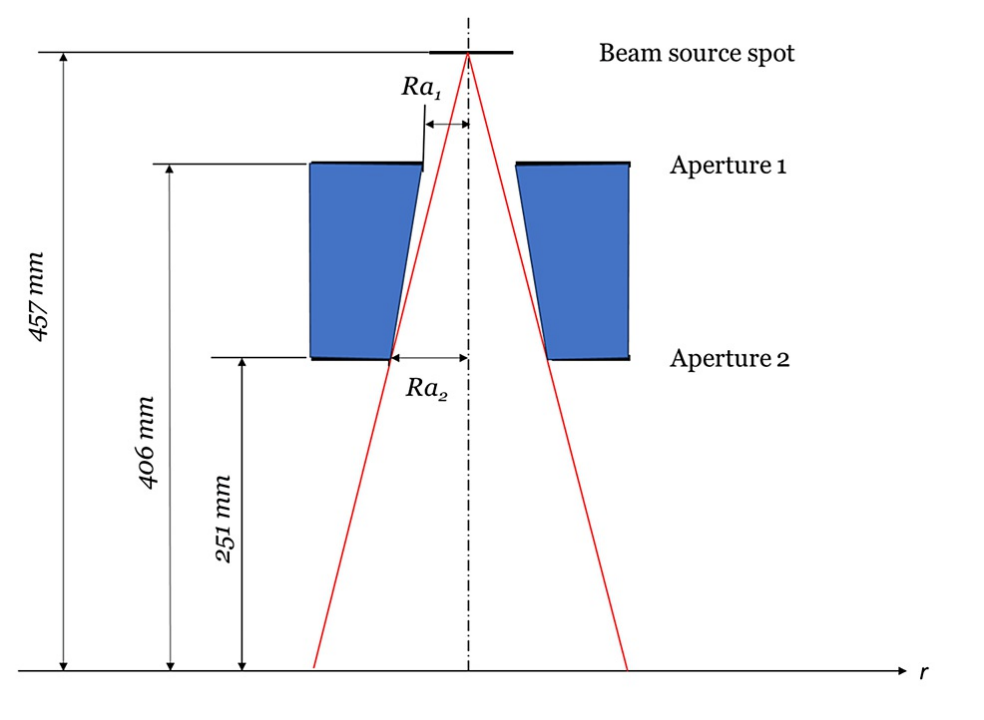
The schematic of the example collimator system

The schematic of the collimator system is shown in Figure 3. For simplicity, cylindrical symmetry is assumed for the system. The beam spot intensity is a Gaussian distribution with an FWHM (full width at half maximum) of 1.5 mm. Assuming that the system has a fairly narrow aperture, the intensity variation with angle $\theta$ is omitted. The conical collimator is represented by two apertures at the top and bottom of the conical shape. The target plane is 457 mm from the target. The collimator is designed to produce a 25 mm diameter field. This structure is a simplified version of the ZAP-X (ZAP Surgical, San Carlos, CA, US) beamline [6]. It is designed so that the result can be compared with other methods.

**Figure 4:**
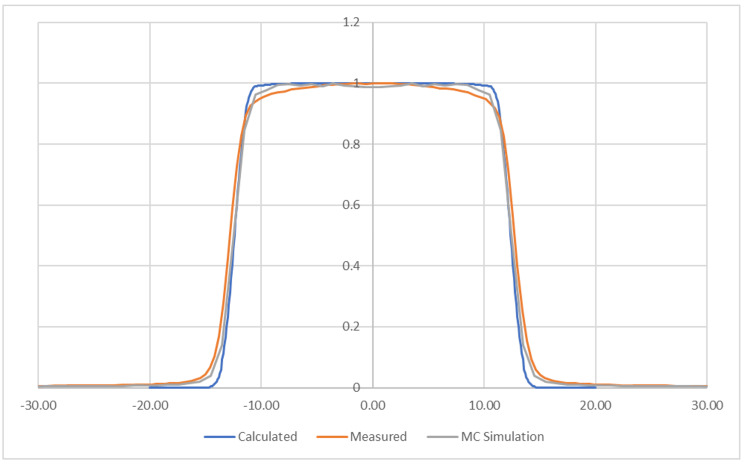
Calculated profile by the projection integration method, comparing with measurement and the Monte Carlo simulation result

The calculated profile is plotted in Figure 4, together with the Monte Carlo simulated profile and measured result. The Monte Carlo result was simulated by Penelope, a well-established Monte Carlo code [7]. The measurement was performed on a ZAP-X system. The data are normalized for comparison. The projection integration method, calculated in less than one second, produced a consistent profile with the Monte Carlo simulation that cost 450 CPU hours on a high-power server.

Table 1 lists the FWHM and the penumbra obtained from the Monte Carlo simulation and the projection integration method in comparison to the measurement. The measured FWHM matches within 4.16%.

**Table 1:** Comparison of beam size and penumbra by different methods FWHM: full width at half maximum

	Measured	MC Simulation	Projection Integration
FWHM (mm)	25.56	24.92	24.5
FWHM difference from measurement		2.50%	4.16%
Penumbra L (20%-80%) (mm)	1.80	1.70	1.34
Penumbra R (20%-80%) (mm)	1.75	1.70	1.34

The general shape of the projection integration method calculation corresponds with both the measured and Monte Carlo computed beam profiles with the largest deviations in the profile shoulder and low-intensity field edge regions. This deviation is most likely caused by the detector volume averaging and the density effects of the used detector that was also applied during the Monte Carlo simulation. The PTW microSilicon diode detector (PTW Freiburg GmbH, Freiburg, Germany), used for this measurement, has an active volume with a diameter of 1.5 mm [8]. The profile calculated with the projection integration method has a 0.1 mm resolution. Therefore, the measured profile has a lower resolution and a rounded shape. The resolution difference also affects the results in Table 1, particularly the penumbra. Other factors contribute to the difference. Since the angular variation is not considered in the calculation, it is likely the cause of the flat top of the beam profile. In addition, both the Monte Carlo simulation and measurements are performed at a 7 mm depth in a water phantom. The lack of radiation scatter in the presented method most likely contributes to the extended lower corners of the profile.

**Figure 5:**
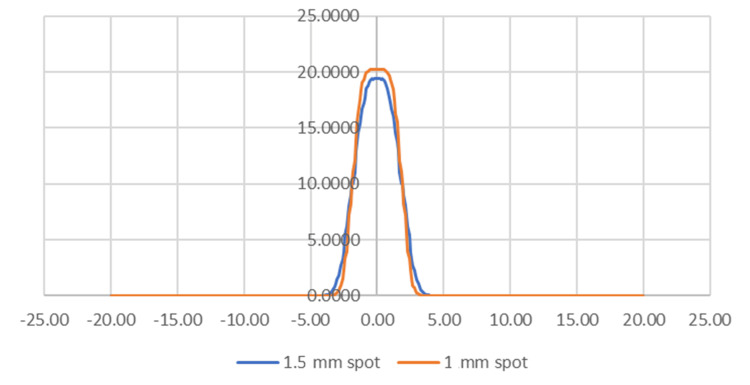
Profiles through the 4 mm collimator calculated by the projection integration method

The projection integration method is an efficient tool to optimize designs of small field pencil beams. One example shown here is a study of beam spot size effects. Figure 5 shows the results of the profile from the 4 mm collimator system. The system is similar to what is illustrated in Figure 3 but has upper and lower aperture diameters of 2.3 mm and 1.8 mm, respectively. The result shows the final profiles for the 1.5 mm and 1 mm beam sources, indicating their impact on the beam profile. While the FWHM values are approximately identical (3.85 vs 3.8 mm, respectively), the penumbras are 1.23 mm versus 0.89 mm. It clearly shows the smaller beam spot generates a smaller penumbra. Additionally, the smaller beam spot also produces a 4% higher peak dose rate. This indicates the necessity of using this tool to optimize the relation between the collimator aperture and the target beam spot size.

## Discussion

The Monte Carlo simulation has been the method used to design and optimize collimators [1]. This method includes most of the physics phenomena. However, the large amount of CPU time required makes it impractical for routine optimization. The advantage of the Monte Carlo method over the method introduced here is that many of the scattering effects are included. As shown above, these effects manifest themselves mostly at the field edge in the shoulder region and the low-intensity region of the beam profile. 

The ray tracing method is widely used in collimator design [2]. This method is fully justified for imaging applications, as it provides point-to-point results. But for beam delivery applications, such as in radiotherapy, the transport of beam emittance is more thorough and efficient than tracing individual particles.

It is worth mentioning that this method is not limited to conical collimators. It can be generalized to rectangular ones as well by calculating orthogonal planes independently or a combing layer-by-layer analysis for multi-leaf collimators (MLC). Further extension of the method can produce 2D distribution. Instead of slicing the intensity in 1D, it can be divided into 2D pixels. Each source pixel projects in every pixel in the treatment plane, resulting in beam intensity in a similar way compared to the above-mentioned method, by integrating over all source pixels.

The beam spot distribution and apertures can be located off-center so that misalignment effects can be studied. This will relate the profile symmetry to the misalignment.

Further details can be added to this method to achieve realistic results. For instance, the projected intensity beyond the aperture may be included with attenuation through the collimator material. This can improve the leakage near the corner of the collimator.

## Conclusions

Complex SRS treatments increase the requirements for more precise design and optimization of beam delivery collimators. They need a fast method to provide the delivered beam profile, beam size, penumbra, as well as dose rate by the collimator system. Traditionally, Monte Carlo simulations are needed to achieve those results. It is well known that Monte Carlo requires intensive computing power and time. It is not suitable for the optimization of designs. Another method being used for collimator design is ray tracing. Faster than the Monte Carlo simulation, ray tracing has to run large numbers of traces to generate a smooth profile. The computing time is still not ideal for design optimizations.

The projection integration method provides a fast, simple tool to design and optimize the collimator to meet tighter requirements, specifically for SRS and small field beams. The method of applying the beam emittance concept instead of individual particles increases the efficiency of calculation drastically and independently of statistical error. It generates a comparable beam profile, beam size, and penumbra as the Monte Carlo simulation, and the measurement magnifies faster than other methods.
